# Investigating the self-assembly of 2NapFF and ureido-pyrimidinone multicomponent systems for cell culture†

**DOI:** 10.1039/d4tb00836g

**Published:** 2024-08-14

**Authors:** Chloe M. Wallace, Maritza M. Rovers, Riccardo Bellan, Martin G. T. A. Rutten, Annela Seddon, Matthew J. Dalby, Patricia Y. W. Dankers, Dave J. Adams

**Affiliations:** a School of Chemistry, University of Glasgow Glasgow G12 8QQ Scotland UK dave.adams@glasgow.ac.uk; b Department of Biomedical Engineering, Institute for Complex Molecular Systems, Eindhoven University of Technology Eindhoven 5600 MB The Netherlands; c School of Physics, HH Wills Physics Laboratory, University of Bristol Tyndall Avenue Bristol BS8 1TL UK; d Centre for Cell Engineering, Institute of Biomedical and Life Sciences, University of Glasgow Glasgow G12 8QQ Scotland UK

## Abstract

Low molecular weight gels are formed *via* the self-assembly of small molecules into fibrous structures. In the case of hydrogels, these networks entrap large volumes of water, yielding soft materials. Such gels tend to have weak mechanical properties and a high permeability for cells, making them particularly appealing for regenerative medicine applications. Ureido-pyrimidinone (UPy) supramolecular gelators are self-assembling systems that have demonstrated excellent capabilities as biomaterials. Here, we combine UPy-gelators with another low molecular weight gelator, the functionalized dipeptide 2NapFF. We have successfully characterized these multicomponent systems on a molecular and bulk scale. The addition of 2NapFF to a crosslinked UPy hydrogel significantly increased hydrogel stiffness from 30 Pa to 1300 Pa. Small-angle X-ray scattering was used to probe the underlying structures of the systems and showed that the mixed UPy and 2NapFF systems resemble the scattering data produced by the pristine UPy systems. However, when a bifunctional UPy-crosslinker was added, the scattering was close to that of the 2NapFF only samples. The results suggest that the crosslinker significantly influences the assembly of the low molecular weight gelators. Finally, we analysed the biocompatibility of the systems using fibroblast cells and found that the cells tended to spread more effectively when the crosslinking species was incorporated. Our results emphasise the need for thorough characterisation at multiple length scales to finely control material properties, which is particularly important for developing novel biomaterials.

## Abstract 2

Low molecular weight gelators have mechanical properties ideal for regenerative medicine. This study explores the combination of a known UPy-system with a dipeptide gelator, 2NapFF, examining each system across multiple length scales.

## Graphical abstract

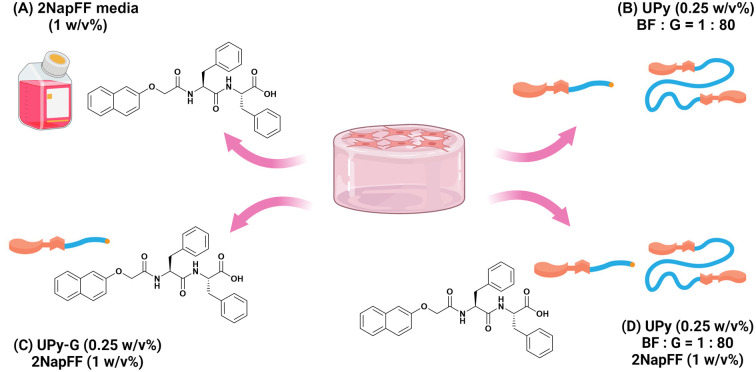

## Introduction

Gels are viscoelastic materials that possess physical properties in between those of a liquid and a solid.^1,2^ They can be classified as either organogels or hydrogels depending on the solvent which is immobilized within the three-dimensional network.^3,4^ Supramolecular hydrogels are a subcategory of hydrogel of particular interest in the fields of bioengineering and regenerative medicine.^5^ In contrast to covalently bonded hydrogels, the self-assembly of supramolecular hydrogels is driven by non-covalent interactions.^6^ This ability to self-assemble non-covalently mimics the hierarchal nature of the extracellular matrix formation, highlighting their potential use for tissue engineering.^7,8^ Furthermore, their highly tunable mechanical properties and degradability have made supramolecular hydrogels of great interest for use in biocompatible scaffolds and encapsulation of bioactive moieties.^5^

**Fig. 1:**
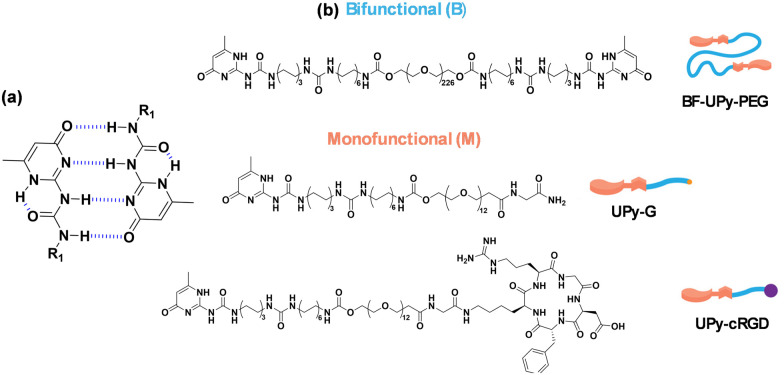
(a) Diagram showing the self-complementary quadruple hydrogen bonding between two UPy-units, where “*R*” represents the respective polymer linker. (b) Structures of bifunctional (B)-type and monofunctional (M)-type molecules as the supramolecular building blocks and additives.

Ureido-pyrimidinone (UPy) based supramolecular polymers are prime candidates for the development of biomaterials for several biomedical applications.^9,10^ The assembly process of these systems is driven by self-complementary UPy–moieties through fourfold hydrogen bonding, which is a relatively strong but reversible interaction (Fig. 1a).^11,12^ This interaction occurs *via* the modification of a hydrophilic prepolymer, *i.e.* poly(ethylene glycol) (PEG) with UPy-units at the chain ends using a hydrophobic alkyl linker and additional urea groups, resulting in the formation of transient aqueous networks and hydrogels.^7,13–15^ These systems are generally composed of two different molecular building block species; monofunctional (M) and bifunctional (B) (Fig. 1b).^7,16^ It has previously been shown that UPy-monomers form one-dimensional fibers, and the bifunctional species act as crosslinker to form transient networks.^7^ Bioactive motifs, typically UPy-cRGD (Fig. 1b), are usually incorporated to enhance the cell adhesion capabilities of the hydrogels by promoting the binding to a number of glycoproteins on the cell surface.^7,16^

Low molecular weight gelators (LMWG), such as monofunctional (M)-type UPy monomers, are a class of non-covalent gelators of interest in the biomedical field.^17,18^ As noted above, these gelators are small molecules that self-assemble into one-dimensional structures, which then entangle into bundles or form a viscoelastic network when a (B)-type crosslinker is present (Mn = 10 kDa, which yields an *n* = 226 on average; BF-UPy-PEG).^19^ Another widely explored type of LMWG is the class of *N*-protected dipeptides. These dipeptides are protected at the *N*-terminus, often with a fluorenylmethyloxycarbonyl (Fmoc) or naphthalene (Nap) group, which contribute to the π–π stacking interactions that promote gelation.^20–22^ Aromatic residues have also been found to affect the elasticity of a gel.^23^ One thoroughly investigated LWMG is 2NapFF, a naphthalene-protected diphenylalanine.^24,25^ This dipeptide amphiphile can form hydrogels using a range of different triggers, including crosslinking *via* divalent cations. This process involves the addition of a divalent metal salt to the gelator solution at high pH, resulting in a metal-coordinated crosslinked gel structure.^26^

Multicomponent systems can be employed to access new properties of a material.^27–30^ This is facilitated by the self-assembly of these systems, leading to the formation of either self-sorted or co-assembled structures.^31,32^ In the case of self-sorted systems, each self-assembled structure only contains one of the components present.^33^ In contrast, in co-assembled systems each structure will contain a mixture of each component.^34^ It is also possible that the properties of each component can be compromised within a multicomponent system. Therefore, fine-tuning each component is required to maximise the benefits of a multicomponent system.

Multicomponent approaches have been reported to prepare supramolecular gels for cell culturing, for example by combining FmocFF with FmocRGD^35^ or FmocS.^30^ As an alternative, here we investigate the multicomponent self-assembly of 2NapFF and UPy-based networks as a complementary approach. Utilising a combined system with UPy building blocks allows for a modular approach, enabling the integration of various UPy functionalities. Furthermore, it has previously been revealed that the addition of different UPy additives to a system can impact cellular response.^36^ We examine these systems across a range of length scales using techniques including small-angle X-ray scattering (SAXS), cryo-transmission electron microscopy (cryo-TEM) and rheology. Finally, we analyze the biocompatibility of the systems using fibroblast cells.

## Results and discussion

**Fig. 2:**
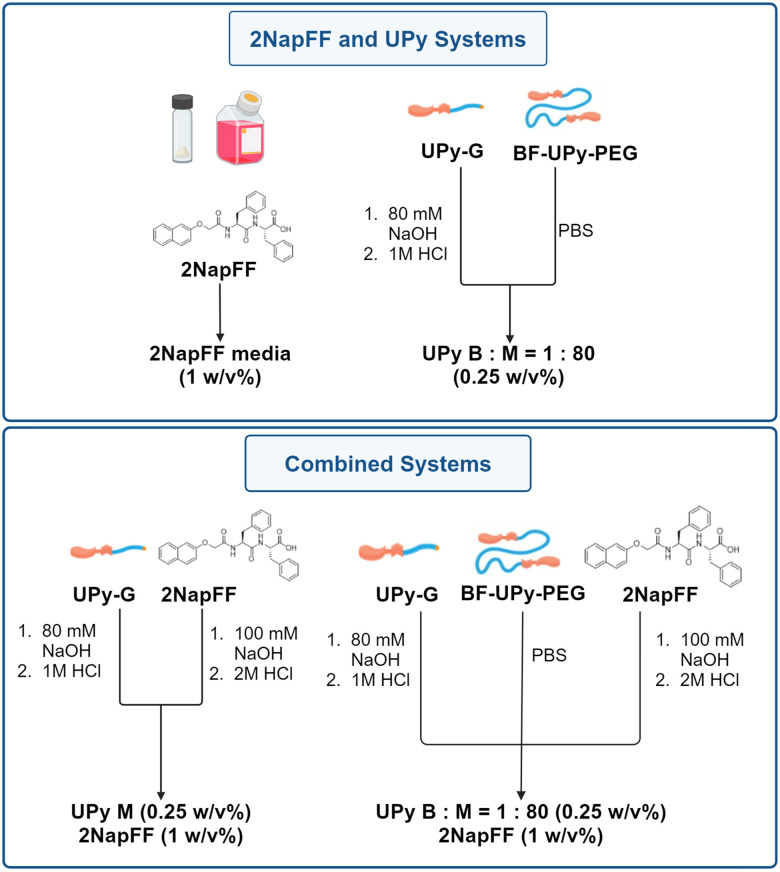
Schematic showing the preparation of the four gel systems used here formed from 2NapFF, UPy-G and BF-UPy-PEG in different combinations. For full experimental details see ESI.†

The gelation of 2NapFF can be triggered by the addition of divalent cations at high pH.^26^ Here, we carry out this crosslinking using Gibco Dulbecco's modified eagle medium (DMEM). The use of culture media allows a physiological pH to be maintained and enhances the biocompatibility of the hydrogels formed. The media also provides a source of cations (including CaCl_2_ and MgSO_4_ divalent ions) that are likely to be triggering the crosslinking of the gelator.^36,37^ As this is the only system involving DMEM, it is important to consider the effect of the cations present on the hydrogel properties. We compared this biocompatible system to that of the UPy-hydrogels and investigated how the gelators can be combined with and without the bifunctional crosslinking species (BF-PEG-UPy) (Fig. 2). For this work, we used the bifunctional UPy-PEG species as a crosslinker and the monofunctional UPy-G (Fig. 1).^7,14^ The UPy components were prepared by dissolving UPy-G in 80 mM NaOH and BF-UPy-PEG in PBS (1X) solution. The ratio of bifunctional to monofunctional monomer used was 1 : 80 (B : M = 1 : 80). The solutions were heated at 70 °C for 1 hour and 30 minutes for the bifunctional and monofunctional respectively before the UPy-G solution was neutralised with 1 M HCl. The solutions were then mixed in the correct proportions to achieve gels at the desired concentrations, before adding this solution to an equal volume of 2NapFF solution at pH 7 if required (Fig. 2). For clarity, we have named these systems A–D as detailed in Table 1.

**Table 1:** Table showing the names of samples (A)–(D) and their corresponding composition

Name	Hydrogel composition
A	2NapFF media (1 w/v%) – 2NapFF crosslinked with Gibco Dulbecco's modified Eagle's medium (DMEM)
B	UPy B : M = 1 : 80 (0.25 w/v%) – bifunctional BF-UPy-PEG species as a crosslinker and the monofunctional UPy-G. The ratio of bifunctional to monofunctional monomer used was 1 : 80.
C	UPy M (0.25 w/v%), 2NapFF (1 w/v%) – monofunctional UPy-G and 2NapFF.
D	UPy B : M = 1 : 80 (0.25 w/v%), 2NapFF (1 w/v%) – BF-UPy-PEG and UPy-G at a ratio of 1 : 80 combined with 2NapFF.

**Fig. 3:**
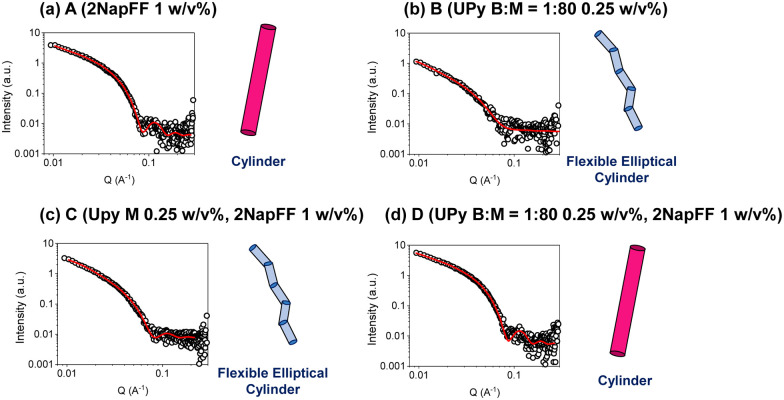
Plots of SAXS data (circles) and fits (red solid lines) along with cartoon (not to scale) of structure represented by fit for (a) A (2NapFF 1 w/v%) (b) B (UPy B : M = 1 : 80, 1 w/v%) (c) C (UPy-M 1.25 w/v%, 2NapFF 1 w/v%) (d) D (UPy B : M = 1 : 80 1.25 w/v%).

To analyze the underlying structures present in these systems, we used small-angle X-ray scattering (SAXS). An advantage of using small-angle scattering over most microscopy techniques is that measurements can be carried out on the solvated, bulk samples.^32^ This eliminates the effect of potential drying artefacts which can interfere with the analysis, as can be the case when using many microscopy techniques.^38^ The SAXS data collected for the 2NapFF gels (A) could be fit best to a cylinder model (Fig. 3a), with the fit showing that the underpinning structures have a radius of 44 Å and a length of 541 Å. This differs from models which 2NapFF hydrogels have been fitted to previously,^25^ and is likely to be due to the neutral pH and salts present in the DMEM buffer. In contrast, the data for the UPy hydrogels (B), fit best to a flexible elliptical cylinder model (Fig. 3b). The UPy structures were considerably longer than the 2NapFF, with a length of over 898 Å, a Kuhn length of 225 Å, minor radius of 36 Å and an axis ratio of 1.7 Å. The C systems also produced data which fit best to a flexible elliptical cylinder model, with similar parameters to that of the pristine UPy samples (Fig. 3c) (Table S1, ESI†). Interestingly, when the bifunctional crosslinking species was introduced, the D hydrogels fit best to a cylinder model with parameters within error of those obtained from the 2NapFF (A) hydrogels (Fig. 3d). To investigate this further, we fit the data for both UPy and 2NapFF mixed systems to a combined cylinder and flexible elliptical cylinder model (Fig. S1, ESI†) (Table S2, ESI†). By setting the parameters to that of the A and B fits respectively and fitting the scales we can gauge the relative contribution of each model to the data.^39^ The C system resulted in a scale of 7.9 × 10^−6^ for the cylinder model and 6.2 × 10^−5^ for the flexible elliptical model. Conversely, the D hydrogels resulted in a 0.003 and 1.4 × 10^−5^ for the cylinder and flexible elliptical cylinder models respectively. This large difference between the two scales suggests that the scattering produced by D is more similar to that of 2NapFF alone (A) than the pristine UPy samples (B). From this observation, we hypothesize that the presence of the BF-UPy-PEG crosslinker leads to self-sorting of UPy and 2NapFF fibers as two separate networks. The highly scattering nature of 2NapFF may dominate the scattering in comparison to the UPy network.^40^ Meanwhile, without the crosslinker, the scale values of the combined models are relatively close (7.9 × 10^−6^ and 6.2 × 10^−5^) with a slightly greater contribution for the flexible elliptical cylinder model. This suggests that without the crosslinking species, the monofunctional UPy-M and 2NapFF undergo coassembly into fibers which resemble those of the pristine UPy. The data and a cartoon model of the scattering objects in each system are shown in Fig. 3.

**Fig. 4:**
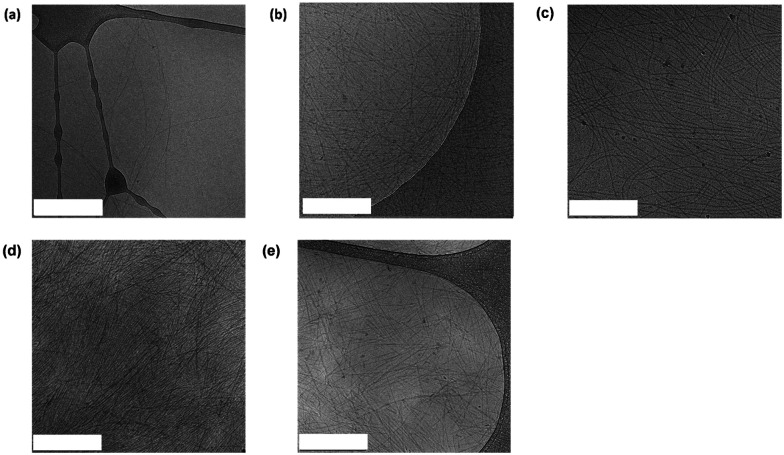
Representative cryo-TEM images for (a) A (2NapFF 1 w/v% hydrogel) (b) UPy M 0.25 w/v% (c) B (UPy B : M 1 : 80 0.25 w/v%) (d) C (UPy M 0.25 w/v%, 2NapFF 1 w/v%) (e) D (UPy B : M 1 : 80 0.25 w/v%, 2NapFF 1 w/v%). The scale bars represent 500 nm in each case.

The materials were then examined using cryo-transmission electron microscopy (cryo-TEM) (Fig. 4). In each case, the images collected revealed long fibrillar structures in agreement with the SAXS data. With respect to the combined systems, C (Fig. 4d) seemed much more densely packed than the other conditions. This also made the individual fibres difficult to analyse. In general, the fibres present in C (Fig. 4d) appear longer than in D (Fig. 4e) which coordinates with the SAXS data collected (Table S1, ESI†). As the fibres present in D were less densely packed we were able to analyse the dimensions of the fibres. From the cryo-TEM images, the average radius of the fibres was calculated to be 42 Å, which is consistent with the SAXS data collected (40 Å). We also calculated the average fibre length to be 1000 Å, which is longer than the length proposed from the SAXS data (525 Å). This discrepancy may be a result of the fibres being outside the range which can be measured by SAXS.

**Fig. 5:**
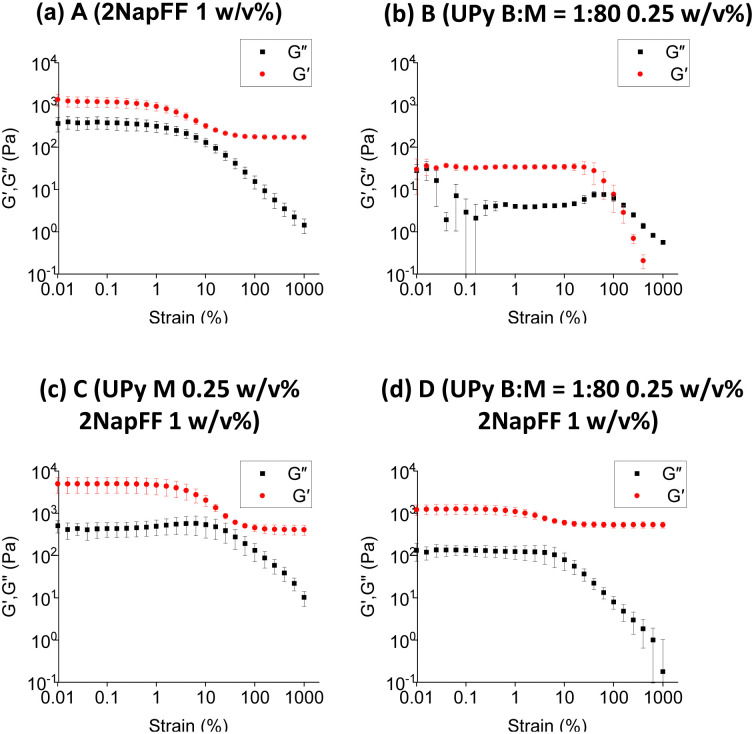
(a)–(d) Strain sweeps of four systems investigated. In each case *G*′ data is red and the storage modulus (*G*′′) is black.

To further understand these systems at larger scales we used oscillatory rheology to probe the bulk material properties at 37 °C. We found the stiffness of the 2NapFF hydrogels (Fig. 5a) to be significantly different to that of the pristine UPy hydrogel, B (Fig. 5b). Firstly, the storage modulus (*G*′) of the 2NapFF (A) samples was significantly higher than the UPy gels. (B) (1300 Pa and 30 Pa respectively). Moreover, the B system has a crossover point at a strain of 100%, whereas the A samples seem to display a drop in *G*′ at 0. 3% strain, before reaching a plateau. Regarding the frequency sweep data, the 2NapFF media system displays an increase in storage modulus at frequencies above 10 rad s^−1^ (Fig. S2a, ESI†). We believe that this is a consequence of the raw phase increasing to 180 °C at higher frequencies, causing the inertia of the rheometer itself to dominate over the hydrogel properties. We observed that the B (Fig. S2b, ESI†) samples produced frequency sweep data with larger error bars compared to the 2NapFF hydrogels. Furthermore, from the corresponding strain sweeps we found that the data points have large errors at strains below 1%. As all the frequency sweeps were carried out at 0.1% strain, we concluded that this combination of low strain and soft material lead to this noisy data. With regards to the multicomponent systems (Fig. 5c and d), there was a slight increase in *G*′ in C (Fig. 5c) compared to when the bifunctional crosslinker was added in D (Fig. 5d). We hypothesise that an interaction is occurring between the 2NapFF and UPy M fibres, which is then disrupted upon the addition of the BF-UPy-PEG crosslinker, due to UPy M having a higher affinity for BF-UPy-PEG. It is possible that when the crosslinker is added the UPy B : M 1 : 80 and 2NapFF form two separate networks, thus D (Fig. 5d) may be the sum of both these networks. To test this theory, we carried out circular dichroism (CD). The 2NapFF DMEM and the 2NapFF UPy-G systems display very similar spectra with a peak at 225–230 nm which denotes π–π stacking of Phe groups present in 2NapFF (Fig. S3, ESI†). When the BF-UPy-PEG was added, the spectrum changed significantly. We also collected data for BF-UPy-PEG and found that it did not produce any signal. This confirms that the change in spectra is due to a change in self-assembly of 2NapFF and UPy M when BF-UPy-PEG is introduced. This self-sorting is consistent with the SAXS data obtained for the system. Another possibility is that due its significantly higher storage modulus, only the 2NapFF component is being measured and the UPy is having little or no influence. This hypothesis is reinforced by the similarities in the *G*′ of A (Fig. 5a) and D (Fig. 5d).

**Fig. 6:**
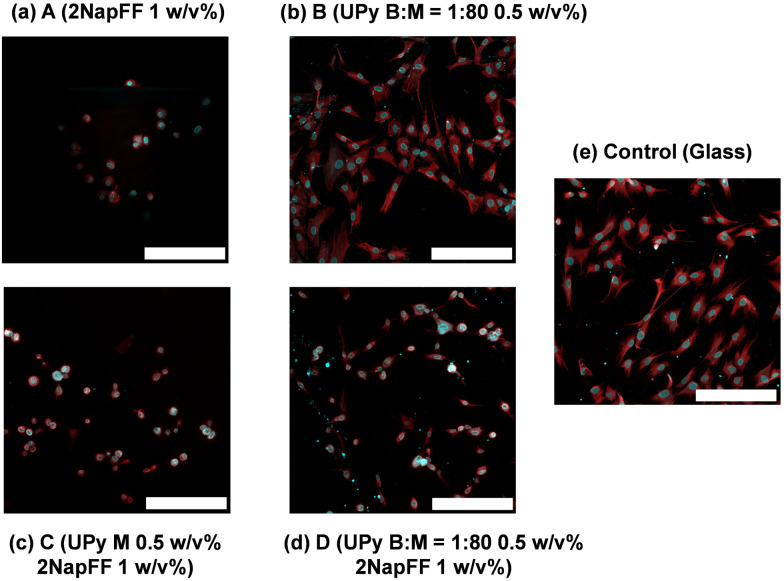
Images of hNDFs 24 hours after seeding on the surface of the hydrogels and stained for nuclei (blue) and actin (red). The cells were seeded on (a) A (2NapFF 1 w/v%) (b) B (UPy B : M = 1 : 80 0.5 w/v%) (c) C (UPy M 0.5 w/v%, 2NapFF 1 w/v%) (d) D (UPy B : M = 1 : 80 0.5 w/v%, 2NapFF 1 w/v%) (e) glass tissue culture plate. For each sample including the UPy-G monomer, 1 mM UPy-cRGD was also included to enhance cell adhesion. Scale bar represents 200 μm in each case.

To understand the biocompatibility of these systems, we cultured fibroblasts in 2D on top of each hydrogel. As previously mentioned, 1 mM UPy-cRGD was incorporated into the UPy-G mixtures both with and without the crosslinker to enhance cell adhesion. Fibroblasts cultured on the 2NapFF hydrogels (A) showed little sign of cell spreading and display a round morphology (Fig. 6a). In contrast, fibroblasts cultured on the UPy only hydrogels (B) adhered effectively and showed clear signs of cell spreading (Fig. 6b). It is proposed that this difference in cell behaviour and morphology is due to the presence of the UPy-cRGD in the UPy system, whereas the 2NapFF gels lack any bioactive species. We concluded that this was a more plausible explanation than a reaction to mechanical stimuli because, despite systems A and D having similar rheological properties (*G*′), the cell morphology observed on the hydrogels varied significantly. Regarding the mixed systems, we noticed that only a few cells adhered to the C hydrogels and those that did exhibit a round morphology and lack of spreading (Fig. 6c). In contrast, cells cultured on the D samples (Fig. 6d) seem to have a morphology more like those cultured on UPy alone B gels (Fig. 6a). From these results, we hypothesize that the UPy-G fibers need to be crosslinked with the bifunctional UPy-PEG molecule to form a network. This is not surprising since pristine UPy-G assemblies are solutions and do not form hydrogels without a crosslinker.^7^ We opted not to carry out a live-dead assay, as our results show as it is the morphology of the cells which indicates if the scaffold is supportive or not. For example, the round cells might stain as live cells, although the material does not support their spreading. Likewise, SEM would offer representative images of the systems as the process requires drying of the samples which leads to morphological changes in such gels.^41^

## Conclusions

Here we have gained a deep understanding as to how two supramolecular systems interact across multiple length scales. By using SAXS to probe the one-dimensional structures of the systems, we were able to deduce that C fits to a flexible elliptical cylinder model with parameters like that of the pristine UPy samples (B). This suggests that the UPy component is driving the self-assembly of these samples, with little contribution from the 2NapFF. Similarly, when the bifunctional crosslinker was incorporated, D fit best to a cylinder model with parameters within error of those obtained from the 2NapFF 1 w/v% hydrogels. We hypothesise that this observation is due to the formation of two sperate networks, with the 2NapFF network dominating the scattering. This is backed up by the oscillatory rheology results which suggest that when BF-UPy-PEG is added to the UPy M and 2NapFF system, two separate networks are formed at a bulk scale. Finally, the 2D cell adhesion studies show that when combining these two supramolecular materials, the BF-UPy-PEG species is essential for crosslinking the UPy-Gly fibers and forming a network to which cells can adhere. These results reveal that, while 2NapFF increases the stiffness of the multicomponent hydrogels, it also limits the adhesive properties of the system Hence, it is essential to consider all factors to fully leverage the advantages of these systems.

Such results reiterate that a change of assembly occurs in the presence of the crosslinker and highlights how understanding such processes can enhance bioactivity. While further optimisation of these systems may be required for cell culture applications, our work reinforces the importance of thorough characterisation of materials. Such characterisation will help us understand and develop finely controllable novel biomaterials in the future.

## Author contributions

Conceptualization: CMW, PYWD, DJA; methodology: CW, MMR, RB, MGTAR, AS, MJD, PYWD, DJA; validation: CMW; formal analysis: CMW; investigation: CW, MMR, RB, MGTAR, AS, MJD, PYWD, DJA; data curation: CW, MMR, RB, MGTAR, AS; visualization: CMW; supervision: PYWD, MJD, DJA; project administration, PYWD, MJD, DJA; funding acquisition, PYWD, MJD, DJA. All authors contributed to the writing of the manuscript.

## Data availability

All data are available in the main text or the ESI† or available on reasonable request.

## Conflicts of interest

There are no conflicts to declare.

## Supplementary Material

TB-012-D4TB00836G-s001
