# Supplementary material for: Investigating the self-assembly of 2NapFF and ureido-pyrimidinone multicomponent systems for cell culture
Source: J Mater Chem B. 2024 Aug 14;12(37):9283–8. doi: 10.1039/d4tb00836g (PMC11340344; doi:10.1039/d4tb00836g)
Supplement: TB-012-D4TB00836G-s001 [file TB-012-D4TB00836G-s001.pdf]

Supplementary Information (SI) for Journal of Materials Chemistry B.
This journal is © The Royal Society of Chemistry 2024

Investigating the self-assembly of 2NapFF and ureido-pyrimidinone multicomponent systems for cell culture

Chloe M. Wallace,[a] *Maritza M. Rovers,*[b] *Riccardo Bellan,*[b] *Martin G.T.A Rutten,*[b] *Annela Seddon,*[c] *Matthew J. Dalby,*[4] *Patricia Y.W Dankers*[b] *and Dave J. Adams*[a]*

a. School of Chemistry, University of Glasgow, Glasgow, G12 8QQ, Scotland, UK

b Department of Biomedical Engineering, Eindhoven University of Technology, Eindhoven, 5600 MB, The Netherlands

c. School of Physics, HH Wills Physics Laboratory, University of Bristol, Tyndall Avenue, Bristol BS8 1TL, U.K.

d. Centre for Cell Engineering, Institute of Biomedical and Life Sciences, University of Glasgow, Glasgow G12 8QQ, Scotland, UK

Supporting Information

Experimental

2NapFF was synthesised as described previously.[1] BF-PEG-UPy, UPy-G and UPy-cRGD, were synthesised by SyMO-Chem and used without further purification. All solvents were of AR quality and purchased from Biosolve. Water was purified on an EMD Millipore Milli-Q Integral Water Purification System.

2NapFF DMEM Gels: A stock solution of 2NapFF (20 mg/mL) was prepared by dissolving the solid gelator in 0.1 M NaOH. This solution was then stirred overnight until a homogeneous solution is observed at pH 10.5. The solutions were then pH adjusted to pH 7 using 2 M HCl. An equal volume of DMEM was quickly pipetted into Eppendorfs containing 2NapFF solution (20 mg/ mL) at pH 7 and vortexed using a vortex mixer, lab dancer VWR for 5 seconds. The gels were then placed in a cell culture incubator overnight at 37°C and 5% CO_2.

UPy-G, BF-UPy-PEG Gels: Solid UPy-G and BF-UPy-PEG powders were weighed and dissolved separately, UPy-G in 80 mM NaOH and BF-UPy-PEG in PBS solution. The ratio of bifunctional to monofunctional monomer used was 1:80 (B: M=1:80). The solutions were heated at 70°C for 1 hour and 30 minutes for the bifunctional and monofunctional respectively. Neutralisation was achieved by adding 1M HCl to the UPy-G solution. The solutions were pipetted together in the correct proportions to achieve gels at the current weight percentage – 0.25, 0.5% or 1 w/v %. The hydrogels were incubated overnight at 37°C and 5% CO_2.

2NapFF and UPy Gels: Multicomponent gels were formed by preparing UPy- gels as described, at double the required concentration, before quickly adding an equal volume of 2NapFF solution. The solutions were pipetted up and down until a homogeneous solution remained and were allowed to gel overnight in the incubator at 37°C and 5% CO_2.

Rheology: Rheological characterisation was carried out using a TA Discovery HR20 or HR 30 (TA instruments) rheometer. Hydrogels were prepared as previously described and pipetting 100 µL of solution into each well of a 96-well plate. Following overnight incubation, the hydrogels were scooped onto the Peltier plate using a spatula. Samples were then analysed using a flat stainless-steel geometry at (diameter 8 mm) at a gap height of 950 µm. Low viscosity silicon oil (47 V 100, RHODORSIL®) was used around the hydrogel to minimize sample drying. Strain sweeps were performed from 0.1-100% strain at an angular frequency of 1 rad/s. Frequency sweeps were carried out at 0.1% strain from 1-100 rad/s. Strain and frequency sweeps were carried out in triplicate and values averaged. Error bars represent the

standard deviation between the replicates. All measurements were carried out at 37°C using a solvent trap to minimise drying.

SAXS: All samples were loaded into 1.5 mm borosilicate glass capillaries (Capillary Tube Supplies Ltd) and sealed using UV curable adhesive (Norland). SAXS data were collected on a Ganesha 300XL instrument (Xenocs) fitted with a Cu Kα source. SAXS data were collected at room temperature over a Q range of 0.007 – 0.25Å-1 for an exposure time of 3600 seconds. All measurements were corrected for transmission and absolute intensity and had the solvent background and empty capillary scattering subtracted before processing. Data were reduced using SAXSGUI, and model fits were performed using SASView 4.0).[2] To calculate the X-ray scattering length densities (SLDs) for all samples, the NIST neutron activation and scattering calculator was used[3] assuming a density of 1.58 g/cm^3 for the LMWG.

Cryo-TEM: Vitrified thin films for CryoTEM analysis were prepared using an automated vitrification robot (FEI Vitrobot Mark IV) by plunge vitrification in liquid ethane. Before vitrification, a 200-mesh copper grid covered with a Lacey carbon film (Electron Microscopy Sciences) was surface plasma treated for 40 seconds using a Cressington 208 carbon coater. CryoTEM imaging was carried out on the Glacios (Thermo Fisher), equipped with a field emission gun (X-FEG), Ceta 16M camera and a Falcon 4i direct electron detector. The microscope was operated at 200 kV acceleration voltage in bright-field TEM at a nominal magnification of 6.500× and a dose rate of 2 $e^-/Å^2\cdot s$; or at 24.000× magnification and a dose rate of 4 $e^-/Å^2\cdot s$; both with a 1s image acquisition time.

Circular Dichroism: CD was measured using a Chirascan VX CD spectrometer (Applied Photophysics Limited, U.K.) using a quartz cell with a 0.01 mm path length and the following parameters: scanning mode, continuous; scanning speed, 120 nm/min and bandwidth, 1 nm. All CD data are presented as ellipticity and recorded in millidegree (mdeg). Absorbance and high tension (HT) spectra were recorded concomitantly with CD spectra. All spectra were recorded in triplicate and averaged.

2D Cell Culture: The human normal dermal fibroblasts (hNDFs) were cultured in DMEM Advanced medium (Gibco) supplemented with 10% fetal bovine serum (FBS), 1% penicillin-streptomycin (P/S), and 1% Gluta MAX at 37 °C and 5% CO_2. 2D cell culture of hNDFS on the hydrogel samples was carried out by preparing the gels in 15-well Ibidi slides (10 µL per well). After overnight incubation the hydrogels were UV-sterilised for 15 minutes, before 30

μL of media was added to the top of each sample and incubated for 1 hour at 37 °C and 5% CO_2. After this, hNDF cells were seeded at a density of 5000 cells per well.

Cell staining and Imaging: After overnight incubation media was removed and the samples were washed three times with PBS. The hNDFs were then fixed 3.7% for 10 minutes whereafter 0.5% Triton X-100 in PBS was added for another 10 minutes to permeabilize the cells. Cells were washed twice with PBS and blocked with 10% goat serum in 0.05% Triton X-100 in PBS for 30 minutes. Next, the cells were incubated with the primary antibodies anti-Lamin A (1:200; Abcam ab26300) and vimentin (1:300; Abcam ab20346) diluted in 2% goat serum in 0.05% Triton X-100 in PBS overnight at 4 °C. Thereafter, the cells were washed three times with PBS and incubated with the secondary antibodies anti-rabbit Alexa 647 (for Lamin A) and anti-mouse Alexa 555 (for vimentin) (both 1:250) at room temperature for 2 hours, whereafter the cells were washed three times with PBS. Imaging was performed using a Leica TCS SP8 X confocal microscope (Leica Microsystems). Images were processed in ImageJ to create max-projection images of the original z-stacks.

Table S1. Summary of the parameters obtained from fitting SAXS data collected for each system. The error of each parameter is shown below each value. Fitting parameters obtained from SasView model fitting of the SAXS data.

	2NapFF 1w/v%	UPy 0.25w/v% B:M = 1:80	UPy 0.25w/v% 2NapFF 1w/v%	UPy B:M =1:80 2NapFF 1w/v%
Model	CYLINDER	FLEXIBLE ELLIPTICAL CYLINDER	FLEXIBLE ELLIPTICAL CYLINDER	CYLINDER
Scale	$0.001 \pm 4 \times 10^{-6}$	135.8 ± 10.6	$0.0004 \pm 1 \times 10^{-5}$	$0.0003 \pm 1 \times 10^{-6}$
Background	$0.004 \pm 1.8 \times 10^{-4}$	$0.006 \pm 2 \times 10^{-4}$	$0.007 \pm 1.8 \times 10^{-4}$	$0.005 \pm 2.2 \times 10^{-4}$
Length / Å	541 ± 26	898 ± 198	738 ± 47	525 ± 23
Kuhn Length / Å		225 ± 58	184 ± 21	
Radius / Å	44 ± 0.1	36 ± 1.7	38 ± 0.6	44 ± 0.1
Axis Ratio		1.7 ± 0.2	1.5 ± 0.06	
X^2	1.52	1.07	1.10	1.33

Table S2. Summary of the parameters obtained when UPy M 0.25 w/v% 2NapFF 1 w/v% and UPy B:M =1:80 0.25 w/v% 2NapFF 1w/v% are fitted to a combined cylinder and flexible elliptical cylinder model. In each case most parameters are fixed and only A Scale (the contribution from the cylinder model) and B Scale (the contribution from the flexible elliptical cylinder model) are fitted. The error of each parameter is stated below each value. Fitting parameters obtained from SasView model fitting of the SAXS data.

	UPy 0.25w/v% 2NapFF 1w/v%	UPy B:M =1:80 2NapFF 1w/v%
Model	CYLINDER + FLEXIBLE ELLIPTICAL CYLINDER	CYLINDER + FLEXIBLE ELLIPTICAL CYLINDER
A Scale	7.9 $\pm$ 2 x 10^{-6}	0.0003 $\pm$ 4 x 10^{-6}
Background	0.005	0.005
A Length / Å	541	541
A Radius / Å	44	44
B Scale	6.2 x 10-5 $\pm$ 1 x 10-6	1.4 x10-5 $\pm$ 2.4 x 10-6
B Length / Å	898	898
B Kuhn Length / Å	225	225
B Radius / Å	36	36
Axis Ratio	1.7	1.7
X^2	1.62	1.23

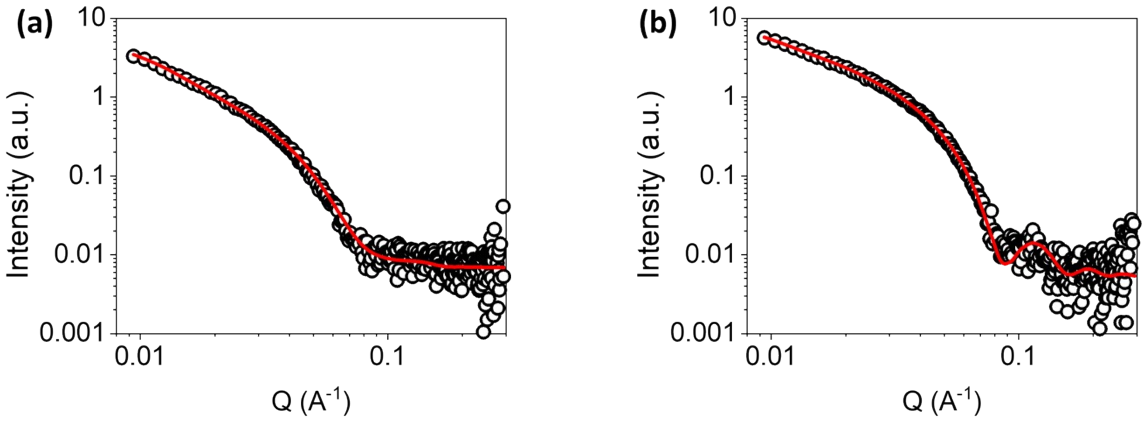

Figure S1. Plots of SAXS data (circles) and fits (red solid lines) for **(a)** UPy M 0.25 w/v% 2NapFF 1 w/v% **(b)** UPy B:M =1:80 0.25 w/v% 2NapFF 1w/v% when fitted to a combined cylinder and flexible elliptical cylinder model. For full parameters see Table S2.

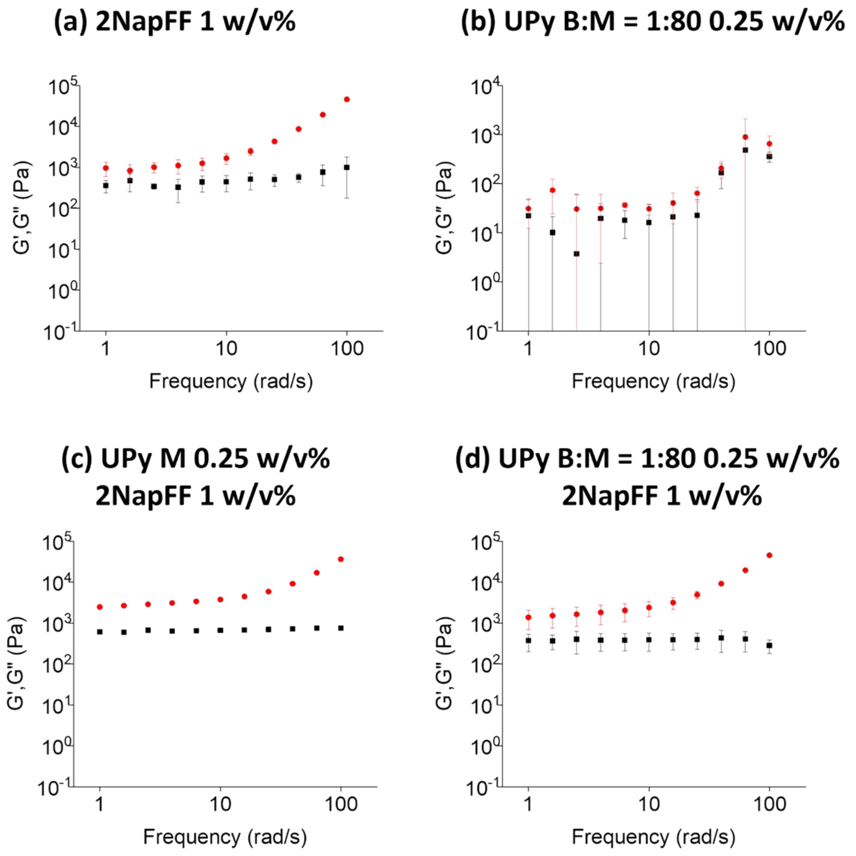

Figure S2. Frequency sweeps of each system. In each case G' data is red and the storage modulus (G") is black.

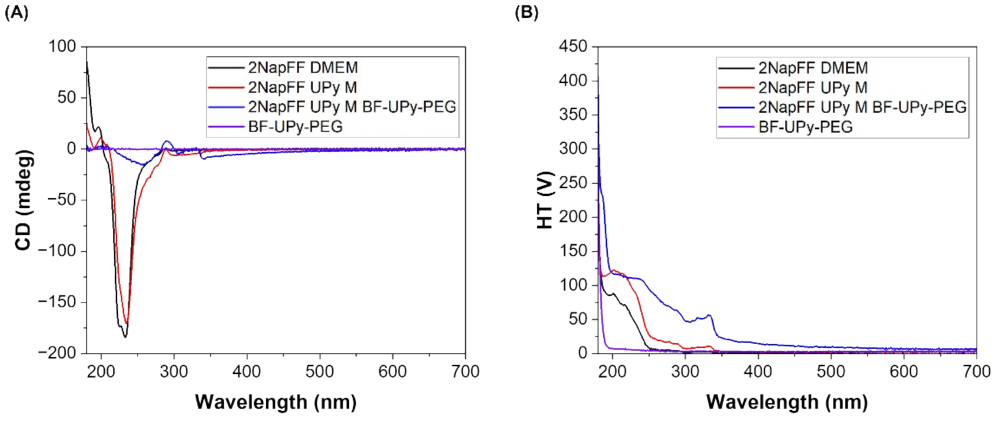

Figure S3. (a) CD and **(b)** HT spectra recorded for systems A, C, D and BF-UPy-PEG alone.

References

1. L. Chen, S. Revel, K. Morris, L. C. Serpell and D. J. Adams, *Langmuir*, 2010, **26**, 13466-13471.
2. https://www.sasview.org/).
3. https://www.ncnr.nist.gov/resources/activation/).
